# Exposure to blood among mortuary workers in teaching hospitals in south-west Nigeria

**Published:** 2012-03-29

**Authors:** Babatunde Ogunnowo, Charles Anunobi, Adebayo Onajole, Kofoworola Odeyemi

**Affiliations:** 1 College of Medicine of the University of Lagos, Nigeria

**Keywords:** Exposure, blood, mortuary workers, infection control, Nigeria

## Abstract

**Background:**

Mortuary workers like other health workers are exposed to blood borne pathogens at work. A baseline assessment is important to plan for programmes to safeguard the health of workers. The aim of this study is to determine exposure rates to blood among mortuary workers in teaching hospitals in South West Nigeria.

**Methods:**

A descriptive cross sectional study was carried out between March and May 2008. All mortuary workers working in six (6) teaching hospitals, 80 in total were included in the study. Data was collected with the aid of a 15- item self administered questionnaire. Data was analysed with the aid of EPI-INFO 2002. Statistical associations were explored using odds ratio and confidence intervals.

**Results:**

A total of 76 respondents completed questionnaire giving a response rate of 95%; 3 males and 1 female declined to participate, the mean age of respondents was 38.2 years, 48(72.6%), 53(85.5%) and 50(73.5%) of the workers had been exposed to blood through cuts, blood splash and needle stick injury. Duration at work was significantly associated with blood splash. Workers who had worked 5years and above were 0.10 times (95% confidence interval 0.00–.0.78) as likely to experience blood splash compared to those who had worked under 5 years. Only 5(10.4%) of workers with needle stick injury had completed three doses of Hepatitis B vaccine. The specific confirmation by antibody titre was however not done in this study.

**Conclusion:**

Exposure to blood was very common with blood splash emerging as the most common route of exposure. There is a need for vaccination of all mortuary workers with three doses of Hepatitis B Vaccine to protect their health. In addition, education of workers on risks and institution of standard operating procedure are crucial to safeguard the health of mortuary workers.

## Background

Mortuary workers like other health workers are exposed to the hazards of blood borne pathogens like Human Immunodeficiency Virus, Hepatitis B and C among others at work. [1,2,3]. Indeed health workers are exposed to up to twenty- seven different blood borne pathogens at work [4]. Previous studies suggest that occupational exposure to blood and body fluids is responsible for 2.5% of Human Immunodeficiency Virus and 40% of Hepatitis B and Hepatitis C among health care workers worldwide [5,6].

In the control of hazards facing health workers, baseline and periodic assessment of exposure to these hazards is an important strategy which is useful as a decision making tool in risk assessment and management of occupational hazards.

There are many studies that focus on exposure assessment to blood borne pathogens among health workers especially nurses and doctors with most studies indicating a higher frequency of exposure to blood and body fluids among nurses [7–9]. Little attention has been paid, however to exposure assessment among mortuary workers even though they are also at risk of contracting diseases from exposure to blood borne pathogens like other categories of health workers.

The objective of this study was to carry out an assessment of exposure to blood among mortuary workers in south west Nigeria. The information obtained will be useful in designing programmes to protect the health of mortuary workers as well as to assess the effect any programme put in place based on gaps identified.

## Methods

A descriptive cross sectional study was conducted among all mortuary workers in six teaching hospitals in South West Nigeria namely the teaching hospitals at Lagos (2), Ibadan (1), Ife (1), Sagamu (1) and Osogbo (1) cities in total there were 80 mortuary workers.

Mortuary workers are often overlooked in studies on health and safety among health workers even though they are also at risk. The majority of mortuary workers in Nigeria work in teaching hospitals, where conditions of service are better, and, as such, are more likely to use best practices. In the city of Lagos, there are some workers in the very few private mortuaries. Mortuary workers in this study include; porters, attendants and others working in mortuary but excludes doctors (pathologists).

The study material was a 15- item questionnaire which was self -administered by the mortuary workers. The study material was in three sections. Section A focused on the socio-demographic characteristics of the workers; section B focused on exposure to blood from various sources while section C focused on Hepatitis B vaccination status. The questionnaire was designed by the investigators based on objectives and results from other related studies. Face and content validation was carried out on study material prior to data collection.

Ethical issues were addressed in the study by the fact that permission was sought from the management of the hospitals as well as the mortuary workers themselves before data was collected. Participation was voluntary and confidentiality of the information obtained was ensured by restricting access of data tools to only the investigators. Ethical approval was not sought from an institutional body because no invasive procedure was carried out during the study.

Data collection took place from March to May 2008. Data was analysed with Epi-info 2002 and presented in tables and figures. In testing association between socio-demographic variables and exposure, odds ratio with 95% confidence intervals were derived.

## Results

A total of 76 completed questionnaires were retrieved and analysed giving a response rate of 95%. Table 1 shows the socio- demographic characteristics of respondents. Most of the respondents were aged between 30–39 years with the mean age being 38.2 years. Almost all (92%) of the respondents were males and (data on faith and tribe was collected as part of socio-demography but was not presented because of limited value and space constraints) married. Majority (69.4%) of the workers were in the junior category and slightly more than one third (36%) had worked for less than 5years. Similarly slightly more than one third (36%) had worked for more than 10 years.

**Table 1:** Socio-demographic characteristics of respondents

Variable	Frequency (%)
**Age group(years) N=76**	
20–29	15(19.7)
30–39	29(38.2)
40–49	21(27.6)
50–59	11(14.5)
**Sex (N=76)**	
Male	69(92)
Female	6(8)
**Educational status(N=73)**	
None	3(4.1)
Primary	24(32.9)
Secondary	24(32.9)
Post-secondary	22(30.1)
**Category of worker(N=72)**	
Senior	22(30.6)
Junior	50(69.4)
**Work duration(N=75)**	
Less than 5years	27(36)
5–10years	21(28)
Greater than 10 years	27(36)

Senior workers are on grade level 8 and above while junior workers are below grade level 8. Education and year of experience play role in category of worker

Table 2 shows the exposure to blood among the respondents in the previous one year prior to data collection. Fifty three workers (85.5%) had been exposed through blood splash. Similarly 50 (73.5%) had experienced needle-stick injury. Exposure to blood through cuts on skin at work occurred in 48(72.7%) of the workers. Exactly half (50 %) of workers had all three exposures. Table 3 shows factors associated with exposure to various forms of exposure to blood.

**Table 2:** Exposure to blood in the past one year through various sources among respondents

Variable	Frequency (%)
Blood splash(N=62)	53(85.5%)
Needle stick injury(N=68)	18(26.5%)
Cuts on skin(N=66)	48(72.7%)
All sources (N=68)	34(50%)

**Table 3:** Factors associated with various sources of exposure to blood among respondents in the past one year

Factors associated with exposure	Needle-stick injury		Blood splash		Cuts on skin	
	Yes (%) odd ratio (95% confidence interval)		Yes (%) odd ratio(95% confidence Interval)		Yes (%) odd ratio (95% confidence interval)	
**Sex of worker**							
Male (N=61)	46(75.4)	3.07(0.36–24.95)	49(80.3)	4.08(0.47–33.59)	44(72.1)	2.59(0.31–20.96)
Female(N=6)	3(50)	1(reference)	3(50)	1(reference)	3(50)	1(reference)
**Category of worker**							
Senior(N=23)	14(66.7)	0.69(0.19–2.59)	16(76.2)	0.62(0.14–2.91)	13(61.9)	0.56(0.16–2.01)
Junior(N= 43)	32(74.4)	1(reference)	36(83.7)	1(reference)	32(74.4)	1(reference)
**Work duration**							
Less than 5years(N=23)	15(65.2)	1(reference)	22(95.7)	1(reference)	16(69.6)	1(reference)
Five years and above(N=45)	35(77.7)	1.87(0.52–6.46)	31(68.9)	0.10(0.00–0.78)	32(71.1)	1.08(0.30–3.63)
**Respondent has ever attended training on safety**							
Yes(N=43)	29(67.4)	0.91(0.25–3.03)	30(69.8)	0.49(0.10–1.91)	28(65.1)	1.20(0.37–3.84)
No (N=23)	16(69.6)	1(reference)	19(82.6)	1(reference)	14(60.1)	1(reference)

With regards to needle-stick injury, males were 3.07 times as likely to experience it compared to females (95% confidence interval of 0.36–24.95). Senior workers were 0.69 times as likely to experience needle-stick injury compared to junior workers (95% confidence interval of 0.19–2.59). Workers who had worked five years and above were 1.87 times as likely to experience needle-stick injury compared to those who had worked under five years(95% confidence interval 0.52 –6.46). In addition, workers who had ever attended a training programme on safety were 0.91 times as likely to experience needle-stick injury compared to those who had not (95% confidence interval 0.25–3.03).

Regarding occurrence of blood splash, males were 4.08 times as likely to experience it compared to females (95% Confidence interval of 0.47–33.59). Senior workers were 0.62 times as likely to experience blood splash compared to junior workers (95% confidence interval of 0.14–2.91). Workers who had worked five years and above were 0.10 times as likely to experience blood splash compared to those who had worked under five years (95% confidence interval of 0.00–0.78,). In addition, workers who had ever attended a training programme on safety were 0.49 times as likely to experience blood splash compared to those who had not (95% confidence interval 0.10–1.91).

With regards to cuts on skin at work, males were 2.59 times as likely to experience it compared to females (95% confidence interval of 0.31–20.96). Senior workers were 0.56 times as likely to experience cuts on skin compared to junior workers (95% confidence interval of 0.16–2.01). Workers who had worked for at least five years were 1.08 times as likely to experience cuts on skin compared to those who had worked under five years(95% confidence interval 0.30 –3.63). In addition, workers who had ever attended a training programme on safety were 1.20 times as likely to experience cuts on skin compared to those who had not (95% confidence interval 0.37–3.84).

Table 4 shows the Hepatitis B vaccination status of workers who had been exposed to needle-stick injury. Among the workers with a history of needle-stick injury, 26(57.8%) had received a dose of hepatitis B vaccine in the past. However, only 5 of the workers with a history of needle-stick injury (10.4%) had completed three doses of hepatitis B required for protection. Similarly among workers with history of blood splash, 29(59.2) had received a dose of hepatitis B vaccine in the past with 8(27.6%) of them completing three doses. Among workers with history of cuts on skin, 22(52.4%) of them had received a dose of hepatitis B vaccine in the past. However, only 3(13.6%) had completed three doses of Hepatitis B vaccine. In all of this, no titre measurement to confirm protection was done.

**Table 4:** Hepatitis B vaccination status of respondents exposed to needle stick injury, cuts and blood splash

Variable	Needle-stick injury Frequency (%)	Cuts Frequency (%)	Blood splash Frequency (%)
**Received a dose of hepatitis B vaccine in the past**			
Yes	26(57.8)	22(52.4)	29(59.2)
No	19(42.2)	20(47.6)	20(40.8)
**Number of doses received (N=26)**			
One	6(23.1)	6(27.3)	6(20.7)
Two	15(57.7)	13(59.1)	15(51.7)
Three	5(19.2)	3(13.6)	8(27.6)

## Discussion

This, to the best of our knowledge, is the first study targeting health and safety of mortuary workers in south west Nigeria with regards to exposure to blood. This is an often overlooked category of workers even though they face risks to their health from exposures at work. Findings from the study will be useful to managers of these institutions, programme managers and all those interested in occupational health and safety.

More than two-thirds of the health workers had been exposed to blood either through cuts, blood splash or needle-stick injury. This finding is much higher than the finding of a study in India with a exposure rate of 32.7% [10], It is also higher than the 43% reported in a study in Iran [11] but is similar to the 79% reported in another study in Southern Iran [12]. In this study, blood splash was the most common form of exposure to blood even though needle-stick injury was also common. This is in agreement with other studies which rank needle-stick injury as a very important source of exposure [13,14].

Exposure through the skin was also common occurring in almost three -quarters of respondents. This necessitates the provision of personal protective devices such as gloves, aprons and boots. Any intervention based on findings of this study will need to focus on availability and utilization of these personal protective devices as well as training on their proper use.

It is worth noting that half of the workers had experienced exposure from all sources indicating the high levels of exposure among workers and implications on their health.

The factors associated with exposure to blood were also explored in this study. Regarding exposures to needle-stick injury, male sex and having worked for more than 5 years was associated with greater exposure to needle-stick injury compared to being female and having worked for less than five years. However, the confidence interval of the odds ratio suggests chance has a role in the findings. Indeed the wide confidence interval suggests that sample size may be inadequate to explore the association. On the other hand, senior workers and those who had attended a training programme on safety were less likely to have needle-stick injury compared to those who were junior and those who had neverattended any training programme on safety. However, chance may explain findings as shown from confidence intervals.

Regarding exposure to blood splash, males were more likely to experience it compared to females, even though chance may explain findings. On the other hand, workers who were in senior category and who had attended a training programme were less likely to experience blood splash compared to junior workers and those yet to attend a training programme even though chance may explain findings. Workers who had spent 5 years and more were less likely to experience blood splash and this was statistically significant as shown from the confidence intervals.

This finding of association between work duration and occurrence of blood splash is in keeping with the findings of a study which showed that more experienced workers were less likely to have exposure to blood [15].

Even though males were more likely to experience blood splash, chance may explain the findings. This is in contrast with findings from a study where males were more likely than females to have injury as source of exposure to blood [16]. The small number of females in study may be responsible for the difference seen.

Regarding exposure to cuts on skin, males were more likely than females to experience it though chance may provide an explanation. In addition, workers who had worked for more than five year were more likely to report it compared to those who had worked less than five years even though chance may explain the findings. On the other, senior workers were less likely to experience cuts compared to junior workers even though chance may explain the findings.The sample size may be inadequate to explore associations as seen from wide confidence intervals.

Among workers who had needle-stick injury, cuts, and blood splash only 19.2%, 13.6% and 27.6% respectively had completed the three doses of Hepatitis B required for protection. This is much lower than the findings of a study in China where over two-thirds of workers were immunized against Hepatitis B [17] but is comparable to findings from other West African countries [18]. This has grave implications for the health and safety of the workers. However, confirmation of protection by antibody estimation was not done.

It will be interesting to explore reasons for low percentage of workers who completed all three doses of Hepatitis B vaccine. It may be that the workers were not motivated to complete all doses or that vaccines were not always available. Further research is required in this regard.

This study is the first study to the best of our knowledge on assessment of exposure to blood among mortuary workers. The findings have documented high exposure through blood splash and cuts on skin. The study however, has its limitations. The sample size was inadequate to explore associations and antibody estimation was also not done.

Nevertheless, the findings will be useful in designing interventions to safe guard the health of the workers as well as for medical audit activities.

## Conclusion

In conclusion, this study demonstrates that in this population of mortuary workers exposure to blood was very common. However, majority of workers with needle-stick injury, cuts and blood splash had not received three doses of Hepatitis B vaccine required for their protection. There is a need to vaccinate all mortuary workers with three doses of Hepatitis B vaccine. In addition, education of workers on risk and training programmes on safety at work will be useful in safe guarding health of mortuary workers.
